# Polymeric Nanocapsules Containing Fennel Essential Oil: Their Preparation, Physicochemical Characterization, Stability over Time and in Simulated Gastrointestinal Conditions

**DOI:** 10.3390/pharmaceutics14040873

**Published:** 2022-04-16

**Authors:** Giuseppe Granata, Carla Riccobene, Edoardo Napoli, Corrada Geraci

**Affiliations:** Istituto di Chimica Biomolecolare—C.N.R., Via Paolo Gaifami 18, 95126 Catania, Italy; carlariccobene@gmail.com (C.R.); edoardo.napoli@icb.cnr.it (E.N.)

**Keywords:** fennel essential oil, poly(ɛ-caprolactone), nanoencapsulation, physicochemical characterization, stability/release, effective bioaccessibility

## Abstract

Plant essential oils, a source of biologically active compounds, represent a promising segment in the pharmaceutical market. However, their volatility, hydrophobicity, poor stability, and low toxicity limit direct use in pharmaceutical-related applications. Nanoencapsulation is a technique that allows overcoming these obstacles by improving bioaccessibility and bioavailability. Nanocapsules (NCs) based on biodegradable and biocompatible poly(ɛ-caprolactone) containing *Foeniculum vulgare* Mill. essential oil (FEO), known for its biological activities, were successfully prepared by interfacial deposition of the preformed polymer method. The composition of FEO (*trans*-anethole chemotype) was determined by gas chromatography analyses. The FEO presence inside the NCs was confirmed by nuclear magnetic resonance experiments. The FEO-NCs showed nanometer size (210 nm), low polydispersity index (0.10), negative zeta potential (−15 mV), non-Newtonian rheological behavior, and high efficiency of encapsulation (93%). Moreover, parameters such as FEO-NC particle size, bioactive compound retention, and FEO composition were monitored for 30 days at storage temperatures of 4 and 40 °C, confirming the robustness of the nanosystem. Finally, FEO-NCs were resistant to the simulated gastric digestion and showed an effective bioaccessibility of 29% in simulated intestinal digestion. Based on the results obtained, this FEO-NC nanosystem could find interesting applications in the nutraceutical and pharmaceutical sectors.

## 1. Introduction

Applications of essential oils (Eos) are an emergent area of research and innovation for the pharmaceutical, medical, and perfume industries. In folk medicine, the prevention and treatment of many diseases by EOs have been known for centuries [1]. EOs are constituted by several bioactive metabolites, including terpene and phenolic compounds which have anti-inflammatory, antimalarial, anticancer, antiviral, antimicrobial, antioxidant, and antiatherosclerotic activities [2]. Some of these activities have already obtained careful scientific and medical scrutiny [3,4,5,6]. The effectiveness of EOs is related to their chemical composition, which depends on environmental factors such as geographical origin, climatic conditions, maturation stages, parts of the plant, and extraction methods, as well as on genetic factors [7].

EOs possess high volatility and poor water solubility, and suffer easy degradation in presence of oxygen, light, high temperature, low pH, and gastrointestinal digestion fluids. Nanoencapsulation is a promising technique that allows overcoming these obstacles [8]. Unlike microcapsules, the nanocapsules have a subcellular size, a larger surface area to volume ratio, greater stability, and are potentially able to enhance bioactive concentrations in water-rich phases or liquid–solid interfaces. Furthermore, the nanoencapsulation technique is known to reduce the interaction of bioactive with food ingredients and decrease its adverse effects, improving bioaccessibility and bioavailability. In addition, the nanocapsules can act as reservoir systems with a controlled release of bioactive over time [9,10].

Fennel (*Foeniculum vulgare* Mill.) is a plant belonging to the Apiaceae family (Umbelliferae) widely diffused in Central Europe and in the Mediterranean areas. It is used as flavoring agent in food products and possesses antimicrobial, antioxidant, antithrombotic, antidiabetic, antitumor, and hepatoprotective activities that make it applicable in the treatment of many inflammatory diseases [11]. *Trans*-anethole is the compound most present in the essential oil of fennel (anethole chemotype). As reported by Aggarwal and Shishodia [12], it is able to interrupt the nuclear transcription of factor kappaB that is related to inflammatory diseases including type 2 diabetes [13]. *Trans*-anethole acts in an early step in the cascade of TNF-dependent signal transduction [14]. Moreover, Sheikh et al. [15] reported that *trans*-anethole possesses hypoglycemic properties capable of reversing the altered activities of key enzymes involved in the metabolism of carbohydrates to near normal.

Although fennel essential oil (FEO) has been extensively studied for chemical composition and biological properties, only very few examples are known regarding its nanoformulation. Transdermal nanoemulsions of FEO [16] were prepared by ultrasonic cavitation and assayed on experimental animals. These nanoemulsions showed a prolonged and enhanced antidiabetic effect compared to pure fennel EO. Barradas et al. [17] reported a hydrogel-thickened nanoemulsion containing FEO oil to be useful for the topical application of psoralen. This formulation allowed higher skin penetration and retention of the lipophilic bioactive. Al-Okbi et al. [18] prepared chitosan nanoparticles loaded with fennel EO, with a superior hypoglycemic effect than the original EO when tested on a dyslipidemic rat model.

Considering the importance of FEO oil activity in anti-inflammatory processes and the possibility of increasing its bioaccessibility and bioavailability by means of the nanoencapsulation technique, in the present study we prepared a nanosuspension of poly(ɛ-caprolactone) (PCL) nanocapsules containing FEO oil for potential use in nutraceutical and pharmaceutical fields. We selected PCL, an aliphatic polyester polymer, biodegradable and biocompatible, with a semicrystalline structure, glass transition temperature Tg (about—60 °C), and low melting point temperature (about 60 °C). It is resistant to water, oil, organic solvent, and chlorine, and possesses low viscosity and a short degradation time. In vivo PCL is biodegraded by the action of the lipase enzyme that hydrolyzes the ester linkage to the non-toxic compound 6-hydroxycaproic. Finally, this compound oxidized to 3- acetyl CoA is totally metabolized in the citric acid cycle and eliminated by renal excretion [19,20,21]. Due to the aforementioned characteristics, PCL has been widely explored for biomedical applications. The PCL-based drug delivery system is used especially for lipophilic drugs including Dexamethasone, Ketoprofen, and Paclitaxel [22,23,24]. The use of PCL in surgical absorbable sutures and implants, tissue bioengineering, and antimicrobial and oral vaccine delivery has been successfully reported [20,25,26].

We prepared PCL-based nanocapsules by interfacial deposition of the preformed polymer method [27] to encapsulate FEO. They are formed by the lipophilic core, in which the EO is confined, surrounded by a polymer matrix in turn coated with a non-ionic surfactant. This later confers to nanocapsule hydrophilicity and stability by steric hindrance.

The nanocapsules were characterized for dimension, polydispersity index (PDI), zeta potential, viscosity, encapsulation efficiency, and loading capacity of essential oil. The stability of the nanocapsules containing FEO (FEO-NCs) and the FEO composition were evaluated over time and at various temperatures to simulate storage processes. Finally, tests of simulated gastrointestinal (GI) digestion using specific enzymes were performed to obtain valid information regarding the bioaccessibility of fennel EO in human GI tract compartments.

## 2. Materials and Methods

### 2.1. Materials

Sorbitan monostearate (SM) and poly(ɛ-caprolactone) (PCL) (Mn 45,000) were purchased from Sigma–Aldrich (Milan, Italy); Tween 80 from Fisher Chemical (Fisher Scientific, Geel, Belgium). Pepsin from porcine gastric mucosa, pancreatin from porcine pancreas, and bile extract porcine were purchased from Sigma-Aldrich (Milan, Italy). All chemicals and solvents were of analytic or pharmaceutical grade. FEO-NC suspension was prepared using Water Chromasolv Plus for HPLC solvent (Honeywell Riedel-de-Haën, Seelze, Germany). The essential oil of *Foeniculum vulgare* Mill used in this study is a commercial sample kindly supplied by Rao Erbe (Valverde, Catania, Italy). A standard mix of *n*-alkanes C_9_-C_22_ was purchased by Alltech (Italy).

### 2.2. FEO Characterization by Gas Chromatographic Analyses

#### 2.2.1. Gas Chromatographic (GC) Analysis of FEO

To obtain the chromatographic profile of the essential oil, a Shimadzu GC-17A Gas Chromatograph equipped with a fused silica capillary column (Supelco SPBTM-5 15 m, 0.1 mm, 0.1 mm) and flame ionization detector (FID) was used. The operating conditions, as previously reported [28], were the following: 60 °C for 1 min, 60–280 °C at 10 °C/min, then 280 °C for 1 min; injector temperature 250 °C; detector temperature 280 °C; carrier gas helium (1 mL/min); split mode (1:200); volume of injection 1 µL (4% essential oil/CH_2_Cl_2_ *v*/*v*). The percentages of compounds were determined from their peak areas in the GC-FID profiles.

#### 2.2.2. Gas Chromatograph–Mass Spectrometry (GC-MS) Analysis of Essential Oil

GC–MS analyses were performed on a Shimadzu GCMS-QP5050A. The operating conditions were the same as in the GC-FID analysis. The mass spectrometer parameters were the following: ionization at 70 eV, ion source temperature at 180 °C. Mass spectral data were acquired in the scan mode in the m/z range 40–400. Oil solutions were injected with the split mode (1:96).

#### 2.2.3. Identification of Essential Oil Components

The identity of the components was based on their retention index relative to C_9_–C_22_ *n*-alkanes on the SPB-5 column and computer matching of spectral MS data with those from NIST MS 107 and NIST 21 libraries [29], and the comparison of the fragmentation patterns with those reported in the literature [30].

### 2.3. Preparation of FEO-Loaded Nanocapsules (FEO-NCs)

FEO-NCs were prepared as the previously reported procedure for the preparation of PCL-based nanocapsules containing thyme and oregano essential oils [31] but with a slight change. Briefly, the organic phase, obtained by stirring SM as a dispersant (35 mg), PCL (100 mg), and FEO (310 mg) in acetone (25 mL) at 30 °C, was injected into the aqueous phase containing 75 mg of Tween 80 in 50 mL of pure water. After stirring (10 min, 25 °C) of the mixture, the organic solvent was removed carefully under vacuum (bath at 30 °C, pressure gradually reduced from 500 to 200 mbar in 30 min, from 200 to 90 mBar in 30 min, and then, kept constant at 90 mBar for 10 min) and the FEO-NC suspension (50 mL) was obtained. To ensure complete organic solvent evaporation, a treatment by N_2_ (30 min at atmospheric pressure) was performed. In the same condition, but in absence of essential oil, the empty NC suspension was prepared.

### 2.4. Characterization of FEO-NCs

#### 2.4.1. Particle size, Polydispersity, Zeta Potential, and pH Measurements

Dynamic light scattering (DLS) experiments were performed to determine the mean diameter (Z-average), the polydispersity index (PDI), and the intensity weighted distribution of FEO-NCs colloidal solution previously diluted (1:200, *v/v*) with pure water. Electrophoretic light scattering (ELS) experiments provided the zeta potential (*ζ*) of FEO-NC nanosuspension, diluted (1:200, *v/v*) with pre-filtered (0.45 µm) 10 mM NaCl aqueous solution. DLS and ELS experiments were carried out on a Zetasizer Nano ZS-90, Malvern Instruments, UK at 25 °C, and data were analyzed using Zetasizer Version 7.02 software. The Z-average and PDI were determined by cumulant analysis according to an ISO method (ISO13321:1996 or ISO22412:2008). The distribution size curves were obtained using a non-negative least squares (NNLS) algorithm.

pH measurements of the FEO-NC suspension were determined by a SevenCompact pH Meter (Mettler Toledo, Milan, Italy) at 25 °C, without previous dilution.

#### 2.4.2. Encapsulation Efficiency (EE) and Loading Capacity (LC) of FEO-NCs

The total content of FEO essential oil (FEO_tot_) in FEO-NC suspension was estimated by UV using an 8453 UV-visible spectrophotometer (Agilent Technologies), and 100 µL of FEO-NC suspension were diluted with 900 µL of water. Then, 40 μL of this mixture was added to 2 mL of acetonitrile to break the nanocapsules, and the absorbance was recorded at the maximum absorption wavelength (λ_max_ 259 nm), corrected from the very light absorbance due to the empty NC suspension. The amount of FEO was obtained by the calibration curve of pure FEO (R^2^ = 0.9995), plotting the absorbance at 259 nm of five solutions at different concentrations of FEO (from 3.0 to 15.0 µg/mL). The total content of FEO in FEO-NC suspension was 5.4 mg/mL.

For the determination of non-encapsulated free FEO (FEO_free_) an aliquot of the FEO-NC suspension (500 µL) was centrifuged for 45 min at 21,000× *g* using a Heraeus Pico 21 centrifuge (Thermo Fisher Scientific, Waltham, MA, USA), the supernatant re-centrifuged (15 min at 21,000× *g*), and then supernatant 40 µL was diluted with 2 mL of acetonitrile. The absorbance of the resulting solution was recorded at 259 nm.

The encapsulation efficiency was calculated using the following Equation (1):

(1) $$EE\;(\%)=\frac{\left[FEO\;encapsulated\right]}{[FEO{]}_{tot}}\times 100$$

where the [*FEO encapsulated*] = [*FEO*]_*tot*_ − [*FEO*]*_free_* represents the amount of *FEO* into *FEO-NC*.

The loading capacity was calculated by the following Equation (2):

(2) $$LC\%=\frac{mass\;of\;FEO\;encapsulated\;}{mass\;of\;FEO-NC}\times 100$$

#### 2.4.3. NMR Experiments on FEO-NCs

^1^H-NMR spectra were acquired on a Bruker Avance 400 spectrometer (400.13 MHz ^1^H; 100.6 MHz ^13^C). Chemical shifts (δ) are expressed in ppm with respect to the partially deuterated water (HOD) signal (4.72 ppm). The sample for NMR experiments was prepared as follows: 500 µL of the FEO-NC suspension was centrifuged (45 min at 21,000× *g*) and the supernatant was removed. The suspension obtained by adding 50 µL of deuterium oxide (D_2_O) to the residue was centrifuged (15 min at 21,000× *g*) and the supernatant was discarded. Finally, the residue containing the FEO-NCs was suspended in D_2_O (450 µL) to perform the ^1^H-NMR spectra.

In order to confirm the presence of the essential oil inside the nanostructure, ^1^H-NMR spectra were carried out on the FEO-NC suspension in D_2_O by adding increasing amounts of acetone-*d*_6_ until reaching a 1:1 ratio with D_2_O.

#### 2.4.4. Rheological Measurements

Rheological experiments were performed on a CVO rheometer (Bohlin Instruments, Malvern, UK) with 2°/60 mm diameter steel cone–plate geometry, at 25 °C, and the results were analyzed by Bohlin R6.51.03 software. The sample was left to equilibrate for 10 min before testing. The viscosity variation was conducted from a 0.1 to 150 s^−1^ shear rate, after a pre-shearing period of 10 s at 0.1 s^−1^ shear rate.

The consistency index (*K*) and the flow behavior index (*n*) were obtained by fitting (R^2^ = 0.999) the rheological data by the power law model (Equation (3)):

(3) $$\eta =K\cdot {\dot{\gamma }}^{n-1}$$

where *η* is the apparent viscosity and $\dot{\gamma }$ is the shear rate.

### 2.5. Stability of FEO-NCs

The nanosuspension containing FEO-NCs was divided into glass vials and stored at two different temperatures (4 and 40 °C) for 30 days. The samples were monitored over a period of 30 days to determine physicochemical parameters such as particle size distribution, PDI, zeta potential, *FEO retention* (%), and composition of FEO into NCs. In particular, *FEO retention* (%) was calculated according to the following Equation (4):

(4) $$FEO\;retention\;\left(\%\right)=\frac{{\left[FEO\;encapsulated\right]}_{t}}{{\left[FEO\;encapsulated\right]}_{0}}\times 100$$

where the numerator is the *FEO encapsulated* concentration at the time t and the denominator is *FEO encapsulated* concentration of freshly prepared suspension.

To determine the composition of encapsulated FEO in PCL nanocapsules stored at different temperatures and times, 500 µL of each sample was centrifuged (45 min at 21,000× *g*), and the precipitate was washed two times with 500 µL of water. The sample was suspended with MeOH (500 µL), centrifuged for 5 min at 3500× *g*, and an aliquot was withdrawn to determine the composition of encapsulated FEO by GC (injection volume 5 µL).

### 2.6. Stability of FEO-NCs under In Vitro Simulated Gastrointestinal (GI) Digestion Conditions and Effective Bioaccessibility

In order to ascertain the stability of FEO-NCs in the gastrointestinal tract, an in vitro digestion model system was constructed according to Zhu et al. [32].

#### 2.6.1. Gastric and Intestinal Phases

One mL of FEO-NC suspension was treated with nine mL of simulated gastric fluid (SGF), prepared by dissolving NaCl (2 g/L) in HCl aqueous solution, as described in United States Pharmacopeia (USP 33), and porcine pepsin (30 mg). The resulting mixture (pH 1.6) was gently shaken (100 rpm, 37 °C) for 2 h. After this time, 1 mL of the acidic mixture was withdrawn to perform DLS and ELS analyses. Then, 2.5 mL of an aqueous solution of sodium bicarbonate 0.75 M were added to the remaining mixture to increase the pH to 7.0 in order to stop the pepsin enzymatic reaction. An aliquot (5 mL) of the resulting mixture was treated with 5 mL of the simulated intestinal fluid (SIF), prepared as described in the United States Pharmacopeia (USP 26) by dissolving potassium phosphate monobasic (6.8 g/L) in NaOH aqueous solution, with the addition of porcine pancreatin (15 mg) and porcine bile extract (75.5 mg). The mixture was incubated to 37 °C for 2 h and centrifuged (by ALC centrifuge PK 130, DJB Labcare for 15 min at 2933× *g*). Then, 5 mL of MeOH were added to 5 mL of the supernatant (“micellar phase”) to block enzymatic reactions. All samples (gastric digest, methanol treated micellar phase) were placed in a refrigerator at −20 °C until HPLC analyses.

#### 2.6.2. Physicochemical Characterization of FEO-NCs under Simulated GASTRIC digestion

The Z-average diameter, PDI, the I-weighted distribution, and the zeta potential (ζ) values of FEO-NCs were obtained by DLS or ELS experiments as above described. The gastric digest of FEO-NC suspension (100 or 10 µL) was diluted with 2000 µL of pure water or pre-filtered (0.45 µm) 10 mM NaCl aqueous solution for DLS or ELS, respectively. Both acidic and neutral (treated with sodium bicarbonate) digests were analyzed.

#### 2.6.3. Determination of Trans-Anethole Concentration

*Trans*-anethole is the main component (83.1%) of FEO, and it was taken as a target compound to estimate via HPLC the FEO amount after a simulated gastrointestinal digestion test. Samples for HPLC analysis were suitably treated before injection (see Supporting Material). HPLC was performed on a Dionex HPLC system (P680 pump, ASI-100 autosampler, UVD170U detector, TCC-100 temperature-controlled column compartment), and using a Phenomenex Luna 5 µm C18 reverse-phase column (250 × 4.6 mm). MeOH (A) and 2.5% formic acid in water (B) were used as mobile phase following this elution program: from 25% to 85% A in 15 min, 85% A for 5 min, from 85% to 100% A in 5 min, 100% A for 5 min, flow 0.8 mL/min, T = 25 °C, λ= 259 nm. The highest chromatographic peak in the chromatogram (relative percentage area >90%) was *trans*-anethole. Five solutions containing different concentrations of FEO (from 12.5 to 62.5 µg/mL) were previously injected (10 µL) to verify the linear response and a calibration linear plot (R^2^ = 0.9997) was obtained by plotting the corresponding chromatographic peak areas versus *trans*-anethole concentration. This allowed for quantifying *trans*-anethole in the sample subjected to simulated GI digestion.

The effective bioaccessibility (EB %) of *trans*-anethole was calculated as reported by Fu et al. [33] using the following Equation (5):

(5) $$EB\;\left(\%\right)=\frac{C_{micelle}}{C_{initial}}\times 100$$

where *C_micelle_* and *C_initial_* are the concentrations of *trans*-anethole (FEO major component) in the micelle fraction and in the initial sample, respectively.

### 2.7. Statistical Analysis

Each experiment was replicated at least two times and measurements were performed at least three times. All data are expressed as mean ± standard deviation (SD).

The analysis of variance (ANOVA) followed by mean comparison (Tukey’s test) at a significance level of 0.05 were performed on the experimental stability data (storage time 0–30 days, at 4 and 40 °C).

## 3. Results and Discussion

### 3.1. Volatile Composition of FEO

The numerous studies carried out on the essential oil composition from *Fennel vulgare* Mill have allowed the identification of three different chemotypes: estragole type, anethole/estragole type, and anethole type [34,35]. As an example, in populations of Sicilian wild fennel (Southern Italy), a predominant chemotype of estragole is observed [28]. Other recent studies report an anethole chemotype for Tunisian and Iranian fennel population [36,37].

Moreover, a Moroccan study reported a significant comparison between wild and cultivated samples that shows how the growing conditions, linked to the domestication of wild fennel, considerably reduce the essential oil yield and contribute to the modification of the phytochemical profile [38].

In this study, we selected a commercial anethole chemotype manufactured from plant material. The chemical analyses by GC and GC/MS allowed for the identification of 29 compounds, covering more than 99% of the total oil composition (Table 1). The profile was dominated by *trans*-anethole, which reached 83% of total composition, followed by limonene (4%) and fenchone (4%). The amount of methyl chavicol was very low (estragole).

The choice of commercial FEO *trans*-anethole chemotype ensured the use of standardized phytocomplex with a high percentage of *trans*-anethole, which was the most relevant biologically active component [39], and a much lower percentage of fenchone and estragole. Since the potential toxicity and carcinogenicity of fenchone and estragole is still a matter of debate, this commercial oil is more suitable for pharmaceutical applications [11,40,41,42].

### 3.2. Preparation and Physicochemical Characterization of FEO-NCs

The PCL-based nanocapsules containing FEO were prepared by interfacial deposition of the preformed polymer [27,31,43]. This is a low-energy method that consists of mixing an aqueous phase (containing a surfactant) and an organic phase (containing the polymer, a dispersant, and the essential oil). During mixing, the organic solvent diffuses into the aqueous phase, causing the formation of nanocapsules in a water suspension. This method, also called nanoprecipitation, leads to the formation of nanoscale particles and is easy to perform, inexpensive, highly reproducible, robust, and scalable [44]. The in vivo use of safety PCL nanocapsules containing an active ingredient allows for a reduction of side effects, and an improved pharmacological response with respect to other colloidal formulations as nanospheres or nanoemulsions [45].

#### 3.2.1. Particle Size, Polydispersity, Zeta Potential, and pH Measurements

**Figure 1:**
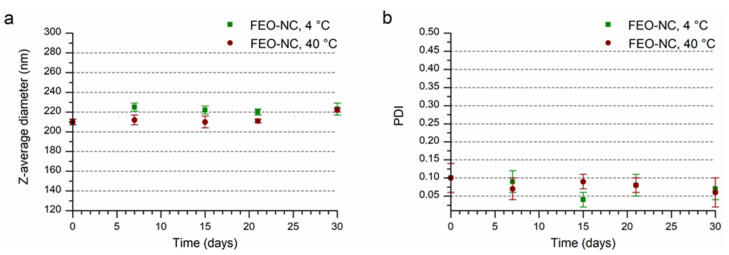
Z-average diameter (**a**) and PDI (**b**) of FEO-NCs over time (storage at 4 and 40 °C).

**Figure 2:**
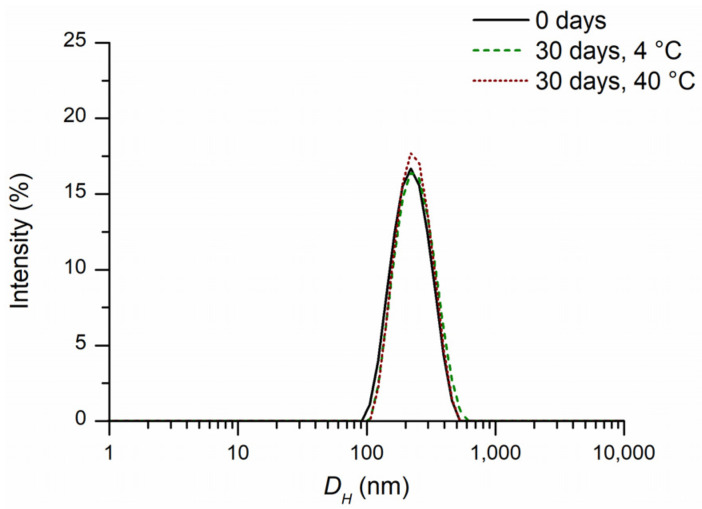
Intensity weighted particle hydrodynamic diameter (*D_H_*, nm) distribution of FEO-NC suspension: freshly prepared, stored at 4 and 40 °C for 30 days.

The value of the principal parameters such as size, PDI, zeta potential, useful to characterize the FEO-NCs are displayed in Tables S1 and S2. The size expressed as the Z-average that represents the intensity weighted mean hydrodynamic size (Figure 1a) was 200 ± 3 nm, confirming the nanometer structure. A narrow size distribution curve (Figure 2, black line) and a low polydispersity index of 0.10 (Figure 1b) were also found. International standards organizations (ISOs) have established that PDI values <0.05 are indicative of highly monodisperse samples, while values >0.7 are ascribable to polydisperse nanoparticle systems (ISO standards ISO 22412:2017 and ISO22412:2017) [46]. Moreover, Patravale et al. [47] reported that a PDI value between 0.1 and 0.25 corresponds to a fairly narrow dimensional distribution of the nanoparticles. Therefore, the FEO-NC suspension can still be considered a nearly monodisperse particle system. This parameter is very important when creating food-grade or pharmaceutical-grade products.

**Figure 3:**
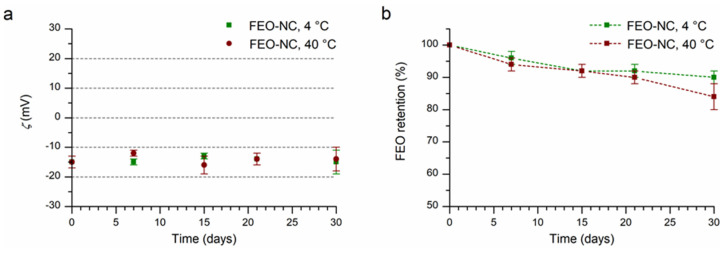
Zeta potential (**a**) and FEO retention (**b**) of FEO-NCs over time (storage at 4 and 40 °C).

The observed zeta potential was low and negative (−15 mV) due to the presence of the Tween 80 (non-ionic surfactant), which surrounds and covers the polymer wall, and the terminal residue acidic groups of the PCL polymeric chain, respectively (Figure 3a). The Tween 80 by steric hindrance confers stability to the nanocapsules in solution. As reported in the literature, there are many examples of PCL nanocapsules [48] containing different bioactives, which possess similar values of zeta potential enough to avoid regrettable phenomena of precipitation and flocculation.

The weakly acidic pH (5.7 ± 0.1) measured for the nanocapsule suspension is attributable to terminal residues of carboxyl groups of the PCL chains.

#### 3.2.2. Encapsulation Efficiency (EE) and Loading Capacity (LC) of FEO-NCs

A high encapsulation efficiency of 93% and notable loading capacity of 53% for FEO-NCs were observed. These interesting data are indicative of the goodness of the encapsulation method, especially in light of a potential large-scale application [48]. In our previous work, we reported similar values for PCL nanocapsules, prepared by the same method, containing oregano and thyme essential oils with 96 and 91 EE%, respectively [31]. To our knowledge, there are no examples of nanoencapsulation of FEO in the literature, while there are only a few examples regarding microencapsulation. An EE of 85% was found for microcapsules of modified starch/chitosan loading fennel oleoresin [49]. The microencapsulation efficiency of fennel EO in different carriers (alginate, chitosan, carrageenan, and carboxymethyl cellulose) ranged between 93% and 98%, as reported by Hussein et al. [50].

Despite these high EE% values reported for microencapsulations, the nanoencapsulation systems characterized by higher surface area and nanoscale range (20–500 nm) are known to possess improved solubility, bioavailability, targeted delivery, and controlled release of the active ingredient [51].

#### 3.2.3. NMR Experiments on FEO-NCs

A preliminary study to confirm the presence of the essential oil inside the FEO-NCs was conducted using NMR spectrometry. Given the complexity of the essential oil, which is a mixture of different volatile compounds, we chose to observe the proton signals of the *trans*-anethole which is the component more abundant in FEO. It is known the NMR spectroscopy is employed to elucidate the structure of organic compounds, as well as to study the molecular interactions that are involved in the formation of supramolecular assemblies such as colloids, liquid crystals, biomolecular condensates, micelles, liposomes, and biological membranes, etc. In these cases, non-covalent bonds hold multiple molecules together.

**Figure 4:**
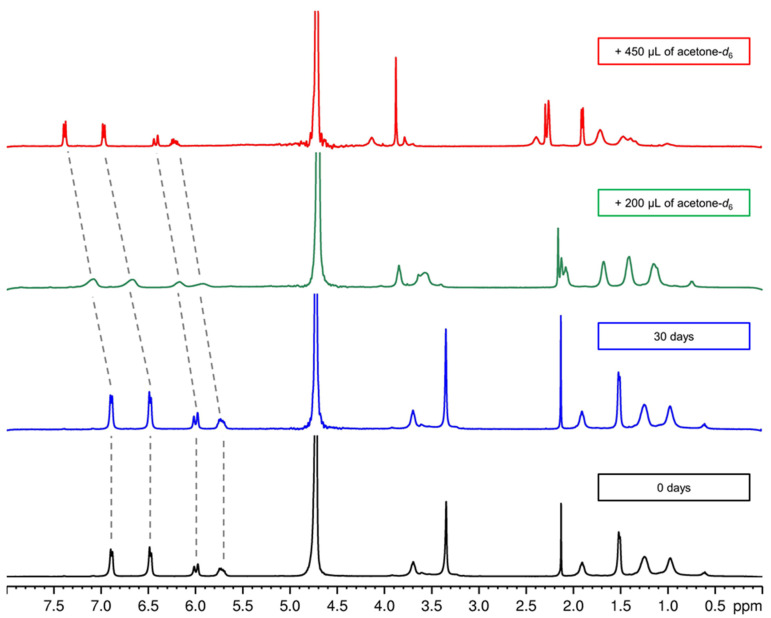
^1^H-NMR of FEO-NC nanosuspension in 450 µL of D_2_O: black line is the freshly prepared sample; blue line is the same sample after 30 days; green line is the sample with the addition of 200 µL of acetone-*d*_6_; red line is the sample with the addition of 450 µL of acetone-*d*_6_.

In particular, we focused our attention on the signal of aromatic ring protons and olefin protons which are located in the region of the NMR spectrum without overlapping signals (Figure 4). For this purpose, the suspension containing the nanocapsules loaded with FEO was centrifuged to remove any residues of free essential oil (not encapsulated). The precipitate was suspended and washed several times in D_2_O to eliminate traces of H_2_O. Finally, an ^1^H-NMR spectrum was performed on the sample suspended in D_2_O.

The spectrum showed two sets of signals for two chemically equivalent aromatic protons at 6.89 (2H, d, J = 7.44 Hz) and 6.48 (2H, d, J = 7.44 Hz) ppm, and two signal at 6.00 (1H, d, J = 15.62 Hz) and 5.63 (1H, m) ppm for olefin protons (Figure 4). ^1^H-NMR spectra were carried out at weekly intervals over a period of 30 days and they showed no change.

To demonstrate that the essential oil is contained within the nanocapsules, we proceeded to add increasing quantities of deuterated acetone to the suspension of FEO-NCs in order to break the nanocapsules and observe the release of the encapsulated essential oil. As expected, after the first additions, the peaks related to the aromatic protons and olefin proton showed a line broadening due to the dynamic process of the spin system [52] and moved to higher ppm values (lower fields). This suggests a transition condition in which the essential oil is partly in the core of the nanocapsule and partly free in the suspension. When the deuterated acetone reaches the 1:1 ratio with respect to the deuterated water, well-resolved peaks are observed for both types of protons belonging to the *trans*-anethole. Moreover, the proton shift at higher ppm values was indicative of a deshielding effect when the EO was free outside the nanocapsule. The data confirm that the essential oil is inside the nanocapsule and constitutes its lipid core. The method we developed using an NMR spectrometer was very simple, easily reproducible in the laboratory, non-invasive, and does not require large quantities of a colloidal dispersion of nanocapsules.

#### 3.2.4. Rheological Measurements

The study of rheological properties of fluids has aroused growing interest related to their applications in various fields of science. Fluid rheological behavior (Newtonian or non-Newtonian) plays an important role in the technological processes mainly related to the food, cosmetic, pharmaceutical, and paint sectors.

**Figure 5:**
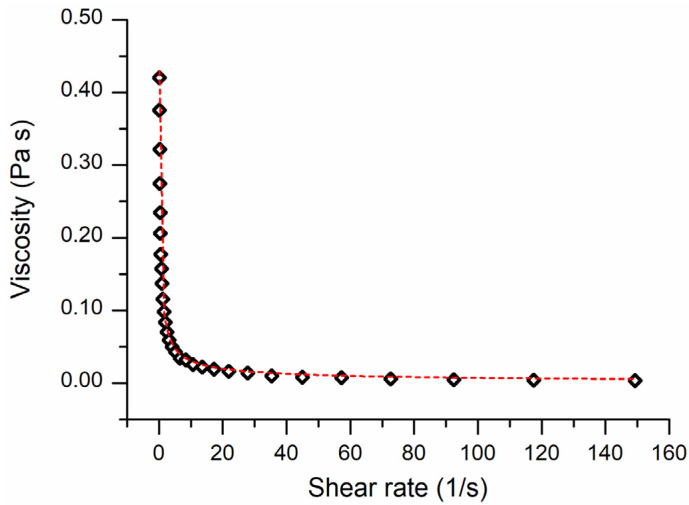
Apparent viscosity variation of the FEO-NC suspension as a function of shear rate. The red dashed line was obtained by fitting the viscosity variation using power law model.

Nanocapsule suspensions are to be considered nanofluids [53] in which nanoparticles with a uniform wall are suspended in a continuous and saturated liquid. The FEO-NC suspensions showed non-Newtonian behavior (Figure 5). The graph was obtained by fitting the rheological data with power law [54] according to the equation reported in Section 2.4.4. The parameters of this equation were the consistency index (K) and flow index (n). K provides information about a fluid viscosity and its dimensions depend on n. When the dimensionless flow index n = 0 the fluid is Newtonian, for n < 1 the fluid is called pseudoplastic when n > 1 dilatant. As shown in Figure 5, the apparent viscosity of FEO-NC suspension goes down to the shear rate increasing indicative of pseudoplastic behavior. The power law model provided values of 0.13 ± 0.01 Pa∙s^n^ and 0.38 ± 0.01 for K and n, respectively (Figure 5).

### 3.3. Stability of the Suspension of FEO-NCs

The suspension of FEO-NCs maintained at 4 or 40 °C for a period of 30 days showed no macroscopic change related to precipitation, aggregation, or phase separation. Instrumental observations also indicated considerable stability during storage, as evidenced by the good overlap of particle size distributions (Figure 2) of the freshly prepared sample and those after 30 days at 4 and 40 °C. The physicochemical measurements (size, PDI, zeta potential, and FEO loaded amount) were reported in Figure 1 and Figure 3 and Tables S1 and S2. The mean hydrodynamic diameter after the first 7 days at a storage temperature of 4 °C showed a slight increase from 210 ± 3 to 225 ± 4 nm (Table S1). The latter value remains almost constant until 30 days of storage. Differently, a slight decreasing trend can be observed for the PD with values ranging from 0.09 ± 0.03 to 0.04 ± 0.02, values considered ideal for stable and monodisperse nanosuspensions [47] (Table S1). The values of zeta potential detected over time were not significantly different (*p* > 0.05). The amount of FEO in nanocapsule suspension of 5.0 ± 0.1 mg/mL gradually decreased until reaching the value of 4.5 ± 0.1 mg/mL at 30 days stored at 4 °C. In the case of the sample kept at 40 °C, a significant difference in mean hydrodynamic diameter after 30 days of storage was observable. However, the found value of 222 ± 2 nm was not such as to affect the stability of the nanocapsules which have PDI (0.06 ± 0.04) typical for stable PCL nanocapsules with narrow size distribution [47]. For the quantity of FEO contained in the nanosuspension, stored at 40 °C for 30 days, a decrease of about 16% compared to the initial one can be observed. This suggests that the nanocapsules act as a reservoir for the essential oil and even under accelerated stability tests, a good amount was still available (Table S2). By extending the storage time to eight months, a drop in FEO of 14% was observed for the refrigerated sample and good dimensional stability was shown (Figure S1).

### 3.4. Composition of Essential Oil in Nanocapsule Suspensions at Different Storage Conditions

The composition of fennel EO in the nanocapsule samples kept under refrigeration (4 °C) and heating (40 °C) conditions for 30 days was evaluated. Gascromatographic analyses were performed at weekly intervals of up to 30 days. As shown in Tables S3 and S4, the percentage of the most abundant components of fennel EO (limonene, fenchone, methyl chavicol, *p*-anisaldehyde, *cis*-anethole, and *trans*-anethole) remained constant in the nanocapsules for up to 7 days in both test conditions. During the whole storage period, a variation in the negligible entity in the relative proportions of the different components such as not to affect the composition of the EO inside the nanocapsules was observed. That finding highlighted the nanoencapsulation effectiveness in protecting essential oil.

**Figure 6:**
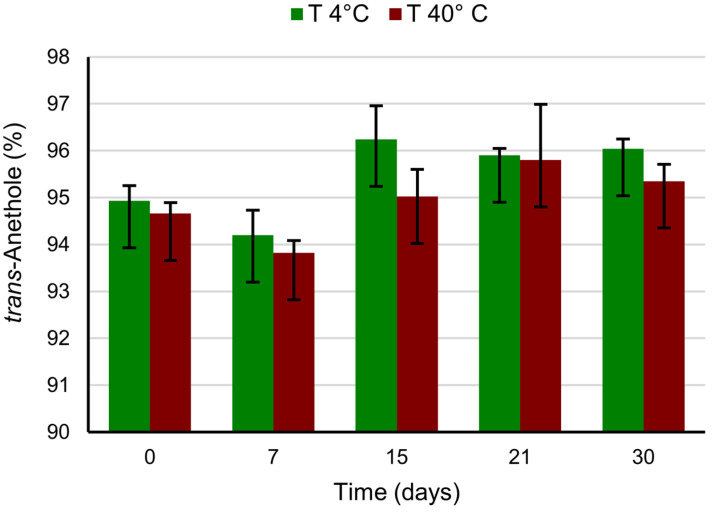
*Trans*-anethole variation by GC-FID (relative percentage) over time and storage at 4 and 40 °C.

In Figure 6, the trend of the *trans*-anethole that is the principal component of fennel EO from time zero (t_0_) up to the end of the storage process is reported. At t_0_, an increase in the relative percentage of *trans*-anethole with respect to FEO before encapsulation was observable, which can be justified by the loss of the minority and more volatile oil components during the preparation phases of the nanocapsules. During storage, the percent values of *trans*-anethole vary within the experimental error due to an imperfect integration of the GC-FID peaks. This is not uncommon when analyzing essential oils with components exceeding 80% of the total composition.

Under refrigerated conditions, *trans*-anethole went from about 95% to 96% after 15 days and remained stable up to 30 days with an increase of about 1%. In heating conditions, the trend was practically the same, going from 94.7% to 95.0% in the first 15 days, and reaching around 95.4% at 30 days.

These findings highlight that nanoencapsulation is highly effective in preventing *trans*-anethole loss during the storage experimental conditions.

### 3.5. Stability Study of FEO-NCs in Simulated Gastrointestinal Medium and Effective Bioaccessibility

The simulated digestion test is widely used to learn about the behavior of food and pharmaceutical products in the gastrointestinal tract. This method attempts to mimic physiological conditions in vivo and has the advantage of being rapid and low cost, and above all does not have the ethical restrictions provided for human trials.

**Figure 7:**
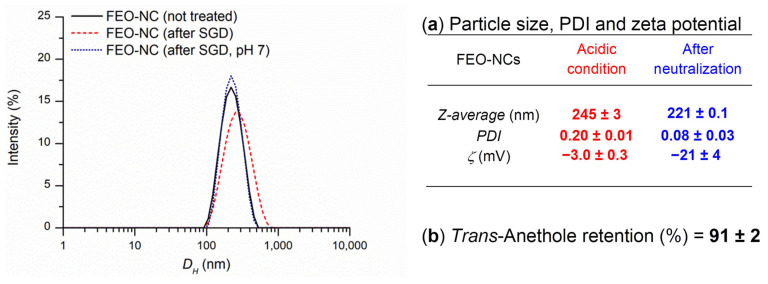
(**Left**) Intensity weighted particle *D_H_* (nm) distribution of FEO-NCs: not treated sample, after simulated gastric digestion (SGD), before and after neutralization. (**Right**) Physicochemical features after SGD (**a**); *trans*-anethole retention percentage after SGD (**b**).

To determine the stability of FEO-NCs after in vitro gastric digestion, physicochemical parameters such as size, PDI, and zeta potential were estimated. As shown in Figure 7, a slight broadening of the peak relative to the hydrodynamic diameter at acid pH was observed. PDI of 0.2 ± 0.01 was indicative of the monodisperse distribution of the digested nanocapsules. The negative zeta potential reached a value of −3.0 ± 0.3 mV, suggesting the protonation of the polymer-free carboxyl groups in a strongly acidic environment. In fact, by neutralizing the digest by adding bicarbonate (pH 7), the zeta potential returned to higher negative values by the deprotonation effect. The Z-average diameter and hydrodynamic distribution of the nanocapsules revert to the observed values before gastric digestion. Given the complexity of the mixture after the simulated gastric digestion, to determine the quantity of *trans*-anethole, FEO’s main component, we decided to measure its percentage retention by HPLC (Figure S2). After 2 h of incubation in the gastric medium, a retention percentage of 91 ± 2 of *trans*-anethole was estimated (Figure 7). This weak release is likely due to the diffusion of non-encapsulated FEO from the polymer matrix surface. The result obtained showed that the polymeric nanocapsules containing FEO were stable in the gastric environment, and the release of the active ingredient was almost negligible.

After gastric digestion, an aliquot of the mixture was used to carry out the simulated intestinal digestion test in the presence of bile and pancreatin for a period of 2 h. The micellar phase was analyzed to determine the effective inaccessibility: a value of 29 ± 0.2% was calculated by Equation (5), as reported in Section 2.6.3. In the simulated intestinal fluid, the release of *trans*-anethole was much faster than in gastric fluid. As reported by Chang et al. [55], PCL polymer degradation in the gastric medium occurs by bulk erosion, while in the intestinal medium an initial non-uniform surface erosion process takes place, followed by hydrolysis of the polymer chain by pancreatic lipase enzymes. These enzymes are capable of dissolving the degraded products, increasing their degradation rate in the intestine.

## 4. Conclusions

In this paper, FEO (*trans*-anethole chemotype) was effectively encapsulated into polymer-based particles using the interfacial deposition technique of preformed polymers. The encapsulation of FEO in PCL polymer nanocapsules was demonstrated by NMR experiments. The excellent physicochemical characteristics (size, PDI, EE, and LC) of the FEO-NCs, even over time (30 days) and at different temperatures (4 °C and 40 °C), suggest that the system is stable and robust. Furthermore, the FEO retention study showed the ability of the PCL nanocarrier to protect it from degenerative phenomena and to act as a reservoir system of essential oil. In both storage conditions, variations of the negligible entity of FEO chemical composition into nanosuspension were observed. The test of the simulated gastrointestinal digestion highlighted the resistance of FEO-NCs in the gastric medium and showed an acceptable effective bioaccessibility of FEO in the intestinal medium.

To our knowledge, this is the first example of fennel essential oil encapsulated in nanocapsules of PCL, a biodegradable and biocompatible polymer.

This green nanosystem could be suitable for the delivery of the FEO and could pave the way for potential applications in various sectors, particularly the pharmaceutical industry.

## Figures and Tables

**Table 1:** Chemical composition of commercial *F. vulgare* EO.

# ^a^	RI lit ^b^	RI Exp ^c^	Class/Compound ^d^	% ^d^
			Monoterpene Hydrocarbons	8.014
1	926	913	tricyclene	0.008
2	930	918	α-thujene	0.009
3	939	926	α-pinene	1.923
4	954	944	camphene	0.102
5	975	970	sabinene	0.033
6	979	974	β-pinene	0.296
7	991	987	β-myrcene	0.288
8	1002	1002	α-phellandrene	0.160
9	1024	1024	*p*-cymene	0.635
10	1029	1030	limonene	4.313
11	1037	1033	*cis*-ocimene	0.178
12	1060	1060	γ-terpinene	0.069
			**Oxygenated monoterpenes**	**4.382**
13	1086	1088	fenchone	4.041
15	1096	1099	linalool	0.128
16	1102	1106	*cis*-thujone	0.039
17	1114	1118	*trans*-thujone	0.006
18	1136	1135	*cis*-limonene oxide	0.008
19	1137	1139	*cis*-mentha-2,8-dien-1-ol	0.016
20	1146	1146	camphor	0.099
21	1152	1154	menthone	0.028
22	1177	1177	terpinen-4-ol	0.018
			**Phenylpropanoids**	**84.744**
23	1196	1194	methyl chavicol	0.583
25	1252	1255	*cis*-anethole	1.040
26	1282	1290	*trans*-anethole	83.121
			**Sesquiterpenes**	**0.089**
28	1417	1405	β-caryophyllene	0.070
29	1434	1419	α-*trans*-bergamotene	0.019
			**Others**	**2.238**
14	1090	1095	methyl benzoate	0.009
24	1250	1248	*p*-anisaldehyde	0.265
27	1382	1370	anysil methyl ketone	1.964
			**TOTAL**	**99.467**

^a^ The numbering refers to elution order; ^b^ literature retention index (RI); ^c^ retention index (RI) relative to standard mixture of n-alkanes on SPB-5 column; ^d^ relative peak area percentages represent averages of 3 determinations.

## Data Availability

Not applicable.

## Supplementary Materials

The following supporting information can be downloaded at: https://www.mdpi.com/article/10.3390/pharmaceutics14040873/s1, Figure S1: Intensity weighted particle *D_H_* (nm) distribution of FEO-NCs: freshly prepared suspension, stored for 50 days, 70 days, and 8 months at 4 °C. Figure S2: Offset HPLC chromatograms showing the peak (time retention 20.6 min) of the FEO main component (trans-anethole) in in vitro simulated digestion experiment: black line sample before the digestion; red line sample after gastric digestion; blue line sample after intestinal digestion. Table S1: Stability over time of fennel essential oil-loaded nanocapsules (FEO-NCs) at 4 °C. Table S2: Stability over time of fennel essential oil-loaded nanocapsules (FEO-NCs) at 40 °C. Table S3: Fennel essential oil composition over time of FEO-loaded nanocapsules at 4 °C. Table S4: Fennel essential oil composition over time of FEO-loaded nanocapsules at 40 °C.
