# Supplementary material for: Polymeric Nanocapsules Containing Fennel Essential Oil: Their Preparation, Physicochemical Characterization, Stability over Time and in Simulated Gastrointestinal Conditions
Source: Pharmaceutics. 2022 Apr 16;14(4):873. doi: 10.3390/pharmaceutics14040873 (PMC9026405; doi:10.3390/pharmaceutics14040873)
Supplement: Supplementary file 1 [file pharmaceutics-14-00873-s001.zip › pharmaceutics-1670163-supplementary.pdf]

pharmaceutics

Supplementary Materials: Polymeric Nanocapsules Containing Fennel Essential Oil: Their Preparation, Physicochemical Characterization, Stability over Time and in Simulated Gastrointestinal Conditions

Giuseppe Granata, Carla Riccobene, Edoardo Napoli and Corrada Geraci

Sample Treating before HPLC Injection for Digest Analysis

After simulated gastric digestion: 1) To determine the total *trans*-anethole amount, 500 µL of acetonitrile was added to 500 µL of (neutral) gastric digest. The mixture, after vortexing, was centrifuged (10 min at 10000 × *g*), and, then, 200 µL of the supernatant was diluted with 800 µL of acetonitrile before HPLC injection. 2) To determine the free *trans*-anethole, the sample was centrifuged at 21,00 × *g* as previously depicted in paragraph 2.4.2., and then 500 µL of supernatant was treated as above described for the total amount. The encapsulated *trans*-anethole amount was obtained by difference (total – free).

After simulated digestion (gastric + intestinal): 500 µL of EtOH was added to 500 µL of the methanol-treated supernatant. The mixture, after vortexing, was centrifuged (10 min at 10,000× *g*), then, 400 µL of the supernatant was diluted with 600 µL of acetonitrile, and, after centrifugation (5 min at 3500 × *g*) an aliquot of this mixture was injected in HPLC.

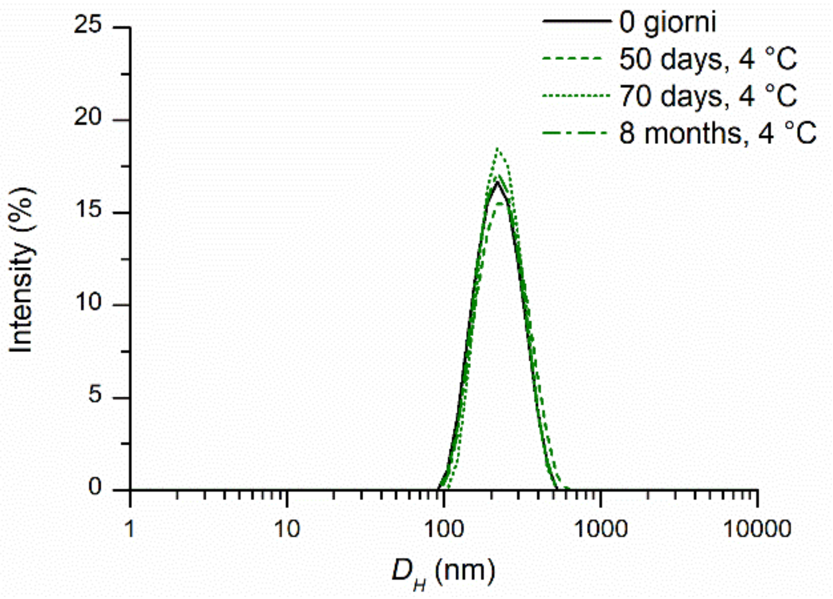

Figure S1. Intensity weighted particle D_H (nm) distribution of FEO-NCs: freshly prepared suspension, stored for 50 days, 70 days, and 8 months at 4 °C.

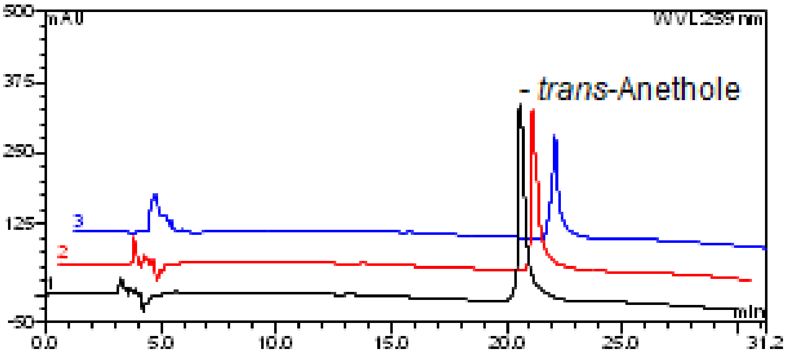

Figure S2. Offset HPLC chromatograms showing the peak (time retention 20.6 min) of the FEO main component (*trans*-anethole) in in vitro simulated digestion experiment: black line sample before the digestion; red line sample after gastric digestion; blue line sample after intestinal digestion.

Table S1. Stability over time of fennel essential oil-loaded nanocapsules (FEO-NCs) at 4 °C.

FEO-NCs	Storage Time (days)				
	0	7	15	21	30
Z-average (nm)	210 ± 3 [a]	225 ± 4 [b]	222 ± 4 [b]	220 ± 3 [b]	223 ± 6 [b]
PDI	0.10 ± 0.04 [a]	0.09 ± 0.03 [a,b]	0.04 ± 0.02 [b]	0.08 ± 0.03 [a,b]	0.07 ± 0.03 [a,b]
ζ (mV)	−15 ± 2 [a]	−15 ± 1 [a]	−13 ± 1 [a]	−14 ± 2 [a]	−15 ± 4 [a]
FEO loaded amount (mg/mL)	5.0 ± 0.1 [a]	4.8 ± 0.1 [a,b]	4.6 ± 0.1 [b,c]	4.6 ± 0.1 [b,c]	4.5 ± 0.1 [c]

Values in the same line with the same superscripts are not significantly different ($p > 0.05$).

Table S2. Stability over time of fennel essential oil-loaded nanocapsules (FEO-NCs) at 40 °C.

FEO-NCs	Storage Time (days)				
	0	7	15	21	30
Z-average (nm)	210 ± 3 [a]	212 ± 5 [a]	210 ± 6 [a]	211 ± 2 [a]	222 ± 2 [b]
PDI	0.10 ± 0.04 [a]	0.07 ± 0.03 [a]	0.09 ± 0.02 [a]	0.08 ± 0.02 [a]	0.06 ± 0.04 [a]
ζ (mV)	−15 ± 2 [a]	−12 ± 1 [a]	−16 ± 3 [a]	−14 ± 2 [a]	−14 ± 4 [a]
FEO loaded amount (mg/mL)	5.0 ± 0.1 [a]	4.7 ± 0.1 [b]	4.6 ± 0.1 [b]	4.5 ± 0.1 [b]	4.2 ± 0.2 [c]

Values in the same line with the same superscripts are not significantly different ($p > 0.05$).

Table S3. Fennel essential oil composition over time of FEO-loaded nanocapsules at 4 °C *.

FEO Component	Storage Time (days)				
(%)	0	7	15	21	30
limonene	0.37 ± 0.03 [a]	0.42 ± 0.07 [a]	0.36 ± 0.03 [a,b]	0.29 ± 0.04 [b]	0.34 ± 0.05 [a,b]
fenchone	0.63 ± 0.05 [a,b]	0.62 ± 0.04 [a]	0.84 ± 0.06 [c]	0.72 ± 0.05 [b]	0.90 ± 0.07 [c]
methyl chavicol	0.40 ± 0.01 [a]	0.40 ± 0.01 [a]	0.45 ± 0.02 [b]	0.44 ± 0.02 [b]	0.44 ± 0.02 [b]
p-anisaldehyde	0.41 ± 0.02 [a]	0.37 ± 0.01 [a]	0.69 ± 0.18 [b]	0.64 ± 0.13 [b]	0.90 ± 0.10 [c]
cis-anethole	0.22 ± 0.07 [a,b]	0.70 ± 0.13 [c]	0.35 ± 0.06 [a]	0.35 ± 0.13 [a]	0.10 ± 0.06 [b]
trans-anethole	94.93 ± 0.23 [a,b]	94.20 ± 0.26 [a]	96.24 ± 0.58 [c]	95.90 ± 1.19 [b,c]	96.04 ± 0.36 [c]

* Components in percentages lower than 0.2 were not considered. Values in the same line with the same superscripts are not significantly different ($p > 0.05$).

Table S4. Fennel essential oil composition over time of FEO-loaded nanocapsules at 40 °C *.

FEO Component (%)	Storage Time (days)				
	0	7	15	21	30
limonene	0.42 ± 0.06 [a]	0.45 ± 0.06 [a]	0.28 ± 0.09 [b]	0.34 ± 0.07 [a,b]	0.38 ± 0.06 [a,b]
fenchone	0.65 ± 0.04 [a]	0.67 ± 0.06 [a]	0.98 ± 0.03 [b]	0.72 ± 0.07 [a]	0.96 ± 0.06 [b]
methyl chavicol	0.40 ± 0.01 [a]	0.42 ± 0.01 [a]	0.45 ± 0.01 [b]	0.45 ± 0.01 [b,c]	0.47 ± 0.01 [c]
p-anisaldehyde	0.39 ± 0.02 [a]	0.48 ± 0.02 [a]	1.15 ± 0.11 [b]	0.87 ± 0.07 [c]	1.29 ± 0.13 [b]
cis-anethole	0.34 ± 0.11 [a]	0.56 ± 0.09 [a]	0.44 ± 0.17 [b]	0.51 ± 0.18 [a]	0.07 ± 0.03 [b]
trans-anethole	94.66 ± 0.32 [a]	93.82 ± 0.53 [b]	95.02 ± 0.72 [a]	95.80 ± 0.15 [c]	95.35 ± 0.21 [a,c]

* Components in percentages lower than 0.2 were not considered. Values in the same line with the same superscripts are not significantly different ($p > 0.05$).
